# miR-99a-5p Regulates the Proliferation and Differentiation of Skeletal Muscle Satellite Cells by Targeting MTMR3 in Chicken

**DOI:** 10.3390/genes11040369

**Published:** 2020-03-29

**Authors:** Xinao Cao, Shuyue Tang, Fei Du, Hao Li, Xiaoxu Shen, Diyan Li, Yan Wang, Zhichao Zhang, Lu Xia, Qing Zhu, Huadong Yin

**Affiliations:** 1 College of Animal Science and Technology, Sichuan Agricultural University, Chengdu 611130, China; caoxinao@stu.sicau.edu.cn (X.C.); tangshuyue@stu.sicau.edu.cn (S.T.); defeichengdu@163.com (F.D.); leo_xixilizi@163.com (H.L.); tiger@sicau.edu.cn (Z.Z.); 2 Farm Animal Genetic Resources Exploration and Innovation Key Laboratory of Sichuan Province, Sichuan Agricultural University, Chengdu 611130, China; shenxiaoxu@stu.sicau.edu.cn (X.S.); diyanli@sicau.edu.cn (D.L.); wangyan519@sicau.edu.cn (Y.W.); xlaza@sicau.edu.cn (L.X.); zhuqing@sicau.edu.cn (Q.Z.)

**Keywords:** miR-99a-5p, SMSCs, proliferation, differentiation, MTMR3

## Abstract

Noncoding RNAs, especially microRNAs (miRNAs), have been reported to play important roles during skeletal muscle development and regeneration. Our previous sequencing data revealed that miR-99a-5p is one of the most abundant miRNAs in chicken breast muscle. The purpose of this study was to reveal the regulatory mechanism of miR-99a-5p in the proliferation and differentiation of chicken skeletal muscle satellite cells (SMSCs). Through the investigation of cell proliferation activity, cell cycle progression, and 5-ethynyl-29-deoxyuridine (EdU) assay, we found that miR-99a-5p can significantly promote the proliferation of SMSCs. Moreover, we found that miR-99a-5p can inhibit myotube formation by decreasing the expression of muscle cell differentiation marker genes. After miR-99a-5p target gene scanning, we confirmed that miR-99a-5p directly targets the 3′ untranslated region (UTR) of myotubularin-related protein 3 (MTMR3) and regulates its expression level during chicken SMSC proliferation and differentiation. We also explored the role of MTMR3 in muscle development and found that its knockdown significantly facilitates the proliferation but represses the differentiation of SMSCs, which is opposite to the effects of miR-99a-5p. Overall, we demonstrated that miR-99a-5p regulates the proliferation and differentiation of SMSCs by targeting MTMR3.

## 1. Introduction

Skeletal muscle is one of the most dynamic and abundant tissues in animals, playing a vital role in movement and metabolism, and accounting for approximately 40% of adult body weight. The amount of skeletal muscle also determines the meat yield of farm animals. The postnatal hypertrophy and regeneration of skeletal muscle mainly depend on adult muscle stem cells named skeletal muscle satellite cells (SMSCs) [1,2]. The process of skeletal muscle satellite cell (SMSC) proliferation and differentiation is also regulated by many signaling pathways, genes, and noncoding RNAs (ncRNAs) [3,4,5].

microRNAs (miRNAs) are a class of endogenous small ncRNAs with a length of 20–24 nucleotides, which generally repress protein synthesis post-transcriptionally by binding to the 3′ untranslated region (3′ UTR) of the target messenger RNAs (mRNAs) [6,7]. An increasing number of studies have shown that miRNAs are involved in skeletal muscle growth and regeneration. For example, miR-1, miR-206, and miR-133 have been identified as being able to regulate skeletal muscle development [8,9]. miR-99a-5p is a member of the miR-99/100 family with the seed region sequence “ACCCGUA”. Previous reports have demonstrated that it mediates cell proliferation, migration, and differentiation by targeting different genes [10,11,12]. However, the function of miR-99a-5p in chicken muscle development is still unknown. Furthermore, in our previous RNA-sequencing study (NCBI Sequence Read Archive database accession number: PRJNA516545), we found that miR-99a-5p is the one of the top five highly expressed miRNAs in chicken embryonic breast muscle, suggesting that it might be a candidate regulator of chicken muscle development. Therefore, in this study, we aimed to gain insight into how miR-99a-5p acts in the regulation of SMSC proliferation and differentiation.

MTMR3 (myotubularin-related protein 3) is an important member of the myotubularin-related protein family. Early studies have shown that MTMR3 has broad homology to myotubularin, including a catalytic domain, and that it also has a C-terminal extension including the FYVE domain, which turns MTMR3 into an inositol lipid 3-phosphatase with unique substrate specificity [13]. In addition, MTMR3 affects cell biological processes by inhibiting autophagy via the repression of mTOR complex 1 (mTORC1) activity [14]. However, until now, it has remained unknown whether MTMR3 plays roles in the growth and hypertrophy of skeletal muscle.

In this study, we investigated the function of miR-99a-5p in the proliferation and differentiation of SMSCs. We found that miR-99a-5p can target MTMR3. We also confirmed the effects of MTMR3 on SMSC proliferation and differentiation in chicken.

## 2. Materials and Methods

### 2.1. Ethics Standards

All animal experimental procedures in this study were approved by the Animal Welfare Committee of Sichuan Agriculture University, and the assurance number is 2019–007.

### 2.2. Animals and Samples

Ross 308 broiler were used in this research and obtained from the Sichuan Yuguan Agriculture Co., Ltd. (Suining, China). All samples collected were quickly frozen in liquid nitrogen and then stored at −80 °C prior to RNA isolation.

### 2.3. Cell Culture

Chicken SMSCs were isolated from the breast muscles of 4-day-old ROSS-308 chickens. Breast muscles were collected, minced, and digested sequentially with 0.2% collagenase type II (Gibco, Langley, OK, USA) and 0.25% trypsin (Gibco) after removing skin and bones. Digestion reactions were terminated by adding equal values of 10% growth medium (GM: Dulbecco’s modified Eagle’s medium (DMEM) (Gibco) + 10% fetal bovine serum (Gibco) + 0.2% penicillin/streptomycin (Invitrogen, Carlsbad, CA, USA)). The suspension was filtered through a cell strainer (pore size: 0.075 mm) and the cells were then isolated by centrifugation. The cells were resuspended in GM and cultured at 37 °C in a 5% CO_2_ humidified atmosphere. Continuously plating was performed to enrich SMSCs and to remove fibroblasts. GM was replaced every day and differentiation medium (DM: DMEM + 2% horse serum (Gibco)) was used to induce SMSC differentiation. DF-1 cells were used for dual-luciferase reporter assay and cultured in 10% GM, and medium was also replaced every 24 h.

### 2.4. RNA Oligonucleotides, Vectors, and Transfection

miR-99a-5p inhibitor, inhibitor negative control (NC), miR-99a-5p mimic, mimic NC, and MTMR3 small interfering RNAs (siRNAs) were synthesized by GenePharma Co., Ltd. (Shanghai, China). Wild-type and mutated sequences of miR-99a-5p binding site in the 3′ UTR region of MTMR3 were synthesized and constructed into pmirGLO vectors (Promega, Madison, WI, USA) using NheI and XhoI restriction sites according to the instructions. All RNA oligonucleotides are listed in Table 1.

At approximately 50% confluence, SMSCs were transfected using Lipofectamine 3000 (Invitrogen) according to the manufacturer’s instructions and cultured in GM to study cell proliferation, whereas cell differentiation was studied in cells transfected at 90% confluence and cultured in DM.

### 2.5. RNA Isolation, cDNA Synthesis, and Quantitative Real-Time PCR (qRT-PCR)

Total RNA was extracted using TRIzol reagent (Takara, Dalian, China) according to the manufacturer’s instructions. cDNA synthesis was performed using One Step miRNA cDNA Synthesis Kit (HaiGene, Haerbin, China, for miRNA) and TransScript One-Step gDNA Removal and cDNA Synthesis SuperMix (TransGen, Beijing, China, for mRNA) according to the manufacturer’s instructions. The qRT-PCR analysis was performed using TB Green PCR Master Mix (Takara), with three biological replicates set for each group. The glyceraldehyde-3-phosphate dehydrogenase (GAPDH) gene was used as internal control. The 2^−ΔΔCt^ method was used to analyze the relative expression level of different qRT-PCR data. The primers are listed in Table 2.

### 2.6. EdU Assay

EdU assays were performed using Cell-Light EdU Apollo567 In Vitro Kits (RiboBio, Guangzhou, China) according to the manufacturer’s protocol. Briefly, SMSCs were seeded in 48-well plates and transfected with siRNAs, mimics, or inhibitors for 48 h. Subsequently, cells were incubated with 50 μM EdU reagent at 37 °C for 2 h, and the cell nuclei were counter-stained with Hoechst 33,342 for 30 min. Three randomly selected fields were captured by a fluorescence microscope (Olympus, Tokyo, Japan). The number of EdU-stained cells was counted by Image-Pro Plus software.

### 2.7. Cell Counting Kit 8 (CCK-8) Assay

SMSCs were seeded in 96-well plates and transfected with siRNAs, mimics, or inhibitors. After 12, 24, 36, and 48 h, the cell vitality was monitored using a Cell Counting Kit-8 kit (Multisciences, Hangzhou, China) according to the manufacturer’s instructions. The absorbance at 450 nm wavelength of each sample was measured using a Microplate Reader (Thermo Fisher, San Jose, CA, USA).

### 2.8. Flow Cytometric Cell Cycle Analysis

SMSCs were seeded in 12-well plates and transfected with siRNAs or vectors for 48 h. cells were collected and suspended in 75% ethanol kept overnight at −20 °C. Then, the cells were incubated with 500 μL Propidium Iodide (PI)/RNase Staining Buffer Solution (BD Biosciences, Franklin Lakes, NJ, USA) at 37 °C for 15 min until detection. Flow cytometric analysis was performed using a BD AccuriC6 flow cytometer (BD Biosciences) and Modfit software.

### 2.9. Luciferase Reporter Assay

DF-1 cells were seeded in 48-well plates and co-transfected with miR-99a-5p mimic or mimic NC and wild-type (WT) or mutated (MT) plasmids. After being transfected for 48 h, cells were lysed and the activities of firefly and Renilla luciferase were measured using Dual-GLO Luciferase Assay System Kit (Promega) with a Fluorescence/Multi-Detection Microplate Reader (Biotek, Shoreline, WA, USA).

### 2.10. Western blot Assay

Detailed experimental methods for Western blot analysis are described in-depth by J. Zhao [15]. Antibodies used for experiments included anti-MyoG (Biorbyt, Cambridge, United Kingdom; diluted 1:1000), anti-MTMR3 (ABclonal, Wuhan, China; 1:2000), and anti-β-tubulin (ZenBio, Chengdu, China; 1:2000). β-tubulin was used as a loading control.

### 2.11. Immunofluorescence Assay

SMSCs were seeded in 24-well plates and cultured in DM and transfected. After 72 h, cells were fixed in 4% formaldehyde. Subsequently, the cells were permeabilized by adding 0.1% Triton X-100 for 20 min and blocked with 5% goat serum (Beyotime) for 30 min. After incubation with anti-Myosin heavy chain (MyHC; Santa Cruz; 1:250) at 4 °C overnight, the cells were then incubated with the Rhodamine (TRITC) AffiniPure Goat Anti-Mouse Immunoglobulin G (IgG; ZenBio; 1:1000) at 37 °C for 1 h. The cell nuclei were stained with 4’,6-diamidino-2-phenylindole( DAPI; Beyotime; 1:50) for 5 min. Three images were obtained at random using a fluorescence microscope (Olympus, Japan). The area of myotubes was measured by Image-Pro Plus software.

### 2.12. Bioinformatic Analysis

miRNA target gene prediction was performed by TargetScan website (http://www.targetscan.org/vert_71/) and miRDB website (http://www.mirdb.org/). Venn analysis was performed by Venn diagrams website (http://bioinformatics.psb.ugent.be/webtools/Venn/).

### 2.13. Statistical Analysis

Data are presented as means ± standard error of the mean (SEM). Significance analysis was performed using SPSS 20.0 (SPSS, Inc., Chicago, IL, USA). Unpaired Student’s *t*-test was used for two group comparison analysis. One-way ANOVA was used for multiple group comparison analysis. *p* < 0.05 was considered as indicating statistical significance.

## 3. Results

### 3.1. Expression Pattern of miR-99a-5p in Chicken

**Figure 1:**
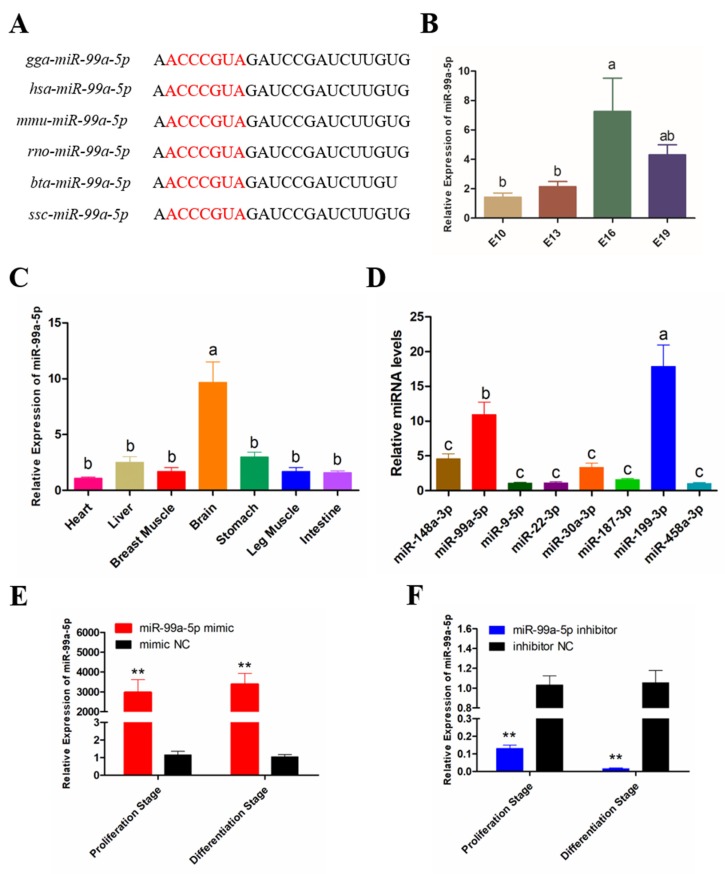
Expression pattern of miR-99a-5p in chicken. (**A**) miR-99a-5p sequence of different species. (**B**) The expression of miR-99a-5p in chicken breast muscle at four embryonic ages. (**C**) The expression of miR-99a-5p in chicken different tissues at embryonic day 16 (E16). (**D**) The expression of eight microRNAs (miRNAs) in chicken breast muscle at E16. (**E**) The transfection efficiency of miR-99a-5p after overexpression of miR-99a-5p. (**F**) The transfection efficiency of miR-99a-5p after inhibition of miR-99a-5p. Results are shown as mean ± standard error of the mean (SEM), and the data are representative of at least three independent assays. One-way ANOVA (**B**,**C**,**D**) and Student’s *t*-test (**E**,**F**) were used to compare expression levels among different groups. * *p* < 0.05; ** *p* < 0.01; ^a,b^ *p* < 0.05.

Sequence analysis revealed that miR-99a-5p is a highly conserved miRNA, the sequence of which is strongly analogous among different species, with only minor differences (Figure 1A). To investigate the expression pattern of miR-99a-5p in chicken skeletal muscle development, qRT-PCR analysis was performed, the results of which showed that miR-99a-5p was differentially expressed during chicken embryonic breast muscle development (Figure 1B). In addition, miR-99a-5p was enriched in chicken brain (Figure 1C), while also being abundant in chicken breast muscle compared with other miRNAs, including the muscle-related miRNAs miR-30a-3p [16] and miR-199-3p [17] (Figure 1D).

SMSCs were isolated and identified by Desmin immunofluorescence staining (Figure S1), then the proliferation and differentiation model were constructed (Figure S2). To investigate the role of miR-99a-5p in SMSC proliferation and differentiation, cells were transfected with inhibitors or mimics at the proliferation stage or differentiation stage. We confirmed that the expression of miR-99a-5p were significantly decreased by the miR-99a-5p inhibitor (*p* < 0.01; Figure 1E), and increased more than 3000-fold in SMSCs by the miR-99a-5p mimic, compared with the levels in the negative control (NC) (*p* < 0.01; Figure 1F), irrespective of whether this was in the proliferation stage or differentiation stage.

### 3.2. miR-99a-5p Promotes the Proliferation of Chicken SMSCs

**Figure 2:**
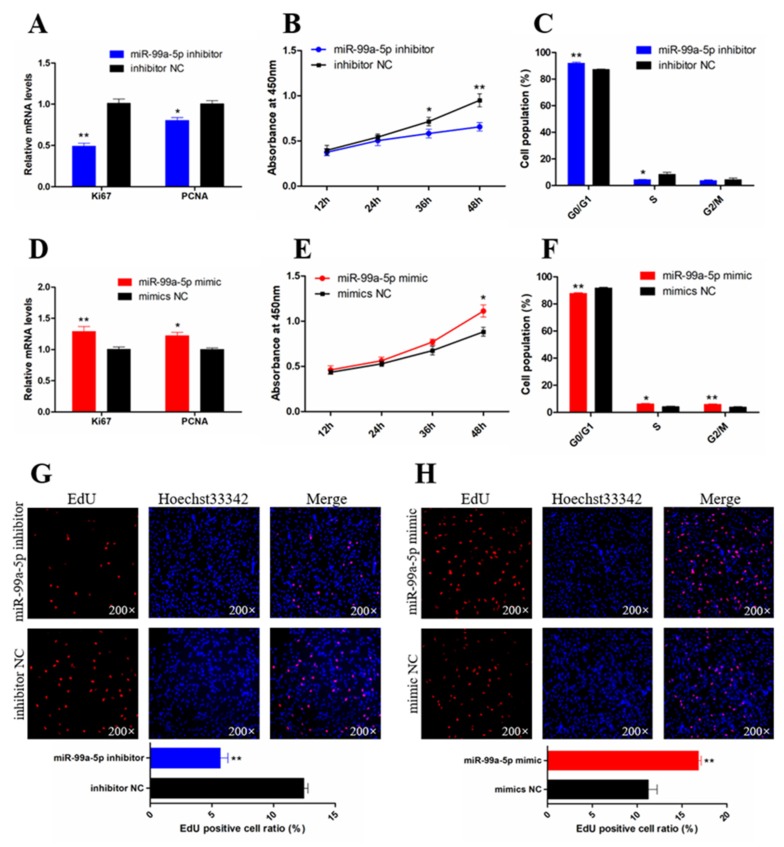
miR-99a-5p promotes the proliferation of chicken skeletal muscle satellite cells (SMSCs). (**A**,**D**) The mRNA levels of cell proliferation-related genes were detected by qRT-PCR in SMSCs after overexpression and inhibition of miR-99a-5p. (**B**,**E**) Cell counting kit 8 (CCK-8) assays for SMSCs after overexpression and inhibition of miR-99a-5p. (**C**,**F**) The statistical results of cell cycle analysis for SMSCs after overexpression and inhibition of miR-99a-5p. (**G**,**H**) EdU staining after the transfection of miR-99a-5p mimic and inhibitor in SMSCs (upper panels). Proliferation rates of chicken SMSCs following miR-99a-5p overexpression and inhibition (lower panels). Results are shown as mean ± SEM, and the data are representative of at least three independent assays. Student’s *t*-test was used to compare expression levels among different groups. * *p* < 0.05; ** *p* < 0.01.

The effects of miR-99a-5p on SMSC proliferation were investigated by qRT-PCR, CCK-8, flow cytometric, and EdU assays. qRT-PCR analysis showed that knockdown of miR-99a-5p significantly decreased the expression of the cell proliferation-related genes marker of proliferation Ki-67 (*Ki67*) and proliferating cell nuclear antigen (*PCNA*) (*p* < 0.05; Figure 2A). CCK-8 assay results showed that SMSC proliferation was significantly inhibited following miR-99a-5p knockdown (*p* < 0.05; Figure 2B). In addition, cell cycle analysis showed that miR-99a-5p knockdown arrested cells in the G_1_ phase (*p* < 0.05; Figure 2C). In contrast, miR-99a-5p overexpression increased the mRNA levels of PCNA and Ki67 (*p* < 0.05; Figure 2D), and also promoted the vitality of SMSCs compared with that in the negative control (*p* < 0.05; Figure 2E). Moreover, miR-99a-5p overexpression promoted the cell cycle progression of SMSCs into the S and G_2_ phases (*p* < 0.05; Figure 2F). The results of the EdU assays also showed significantly reduced SMSC proliferation following miR-99a-5p knockdown (*p* < 0.01; Figure 2G), whereas miR-99a-5p overexpression significantly increased the population of proliferating cells (*p* < 0.01; Figure 2H). Taken together, these results suggested that miR-99a-5p promotes SMSC proliferation.

### 3.3. miR-99a-5p Inhibits the Differentiation of Chicken SMSCs

**Figure 3:**
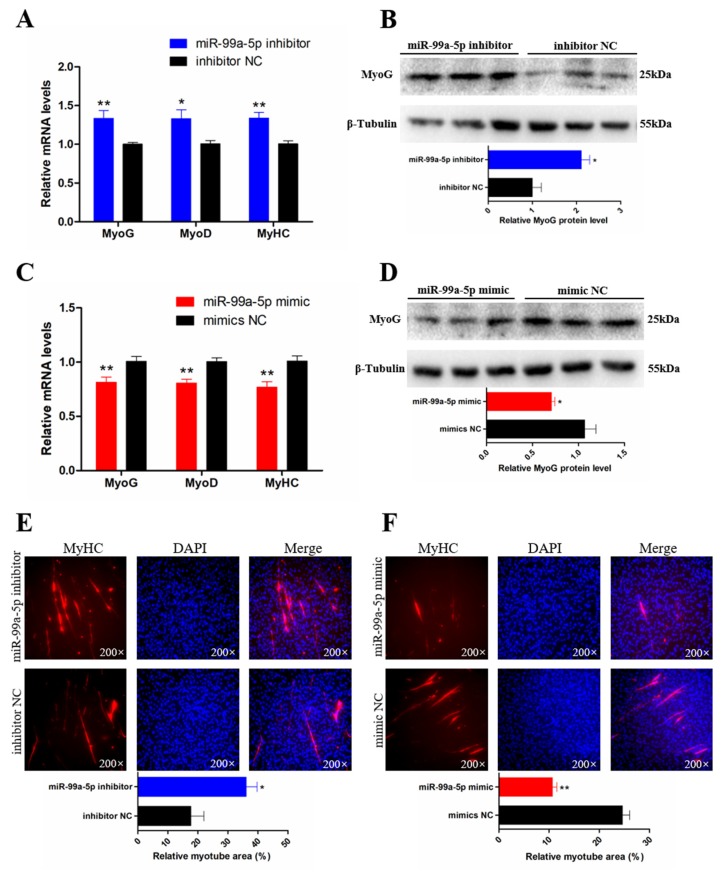
miR-99a-5p inhibits the differentiation of chicken SMSCs. (**A**,**C**) The mRNA levels of muscle cell differentiation marker genes were detected by qRT-PCR in SMSCs after overexpression and inhibition of miR-99a-5p. (**B**,**D**) Western blotting results of myogenin (MyoG) in SMSCs after transfection with miR-99a-5p mimic and inhibitor (upper panels). The relative protein level of MyoG in SMSCs after overexpression and inhibition of miR-99a-5p. (**E**,**F**) Anti-Myosin heavy chain (MyHC) immunofluorescence staining after the transfection of miR-99a-5p mimic and inhibitor in SMSCs (upper panels). Relative myotube area of chicken SMSCs following miR-99a-5p overexpression and inhibition (lower panels). Results are shown as mean ± SEM and the data are representative of at least three independent assays. Student’s *t*-test was used to compare expression levels among different groups. * *p* < 0.05; ** *p* < 0.01.

The ability of miR-99a-5p to regulate SMSC differentiation was investigated by qRT-PCR, Western blot, and immunofluorescence assays. The qRT-PCR analysis showed that the expression of three muscle differentiation marker genes, myogenin (*MyoG*), myoblast determination protein 1 (*MyoD1*), and *MyHC*, was significantly increased following miR-99a-5p knockdown (*p* < 0.05; Figure 3A). Similarly, knockdown of miR-99a-5p also significantly raised the MyoG protein level (*p* < 0.05; Figure 3B). In contrast, overexpression of miR-99a-5p decreased the abundance of RNAs encoded by these three muscle differentiation marker genes (*p* < 0.01; Figure 3C) and the protein level of MyoG (*p* < 0.05; Figure 3D). In addition, MyHC immunofluorescence analysis showed that the relative area of myotubes was significantly increased following miR-99a-5p knockdown (*p* < 0.05; Figure 3E), whereas overexpression of miR-99a-5p decreased the relative area of myotubes compared with that in the negative control group (*p* < 0.01; Figure 3F). These results suggested that miR-99a-5p inhibits SMSC differentiation.

### 3.4. Target Gene Scanning Revealed that miR-99a-5p Directly Targets the MTMR3 Gene

**Figure 4:**
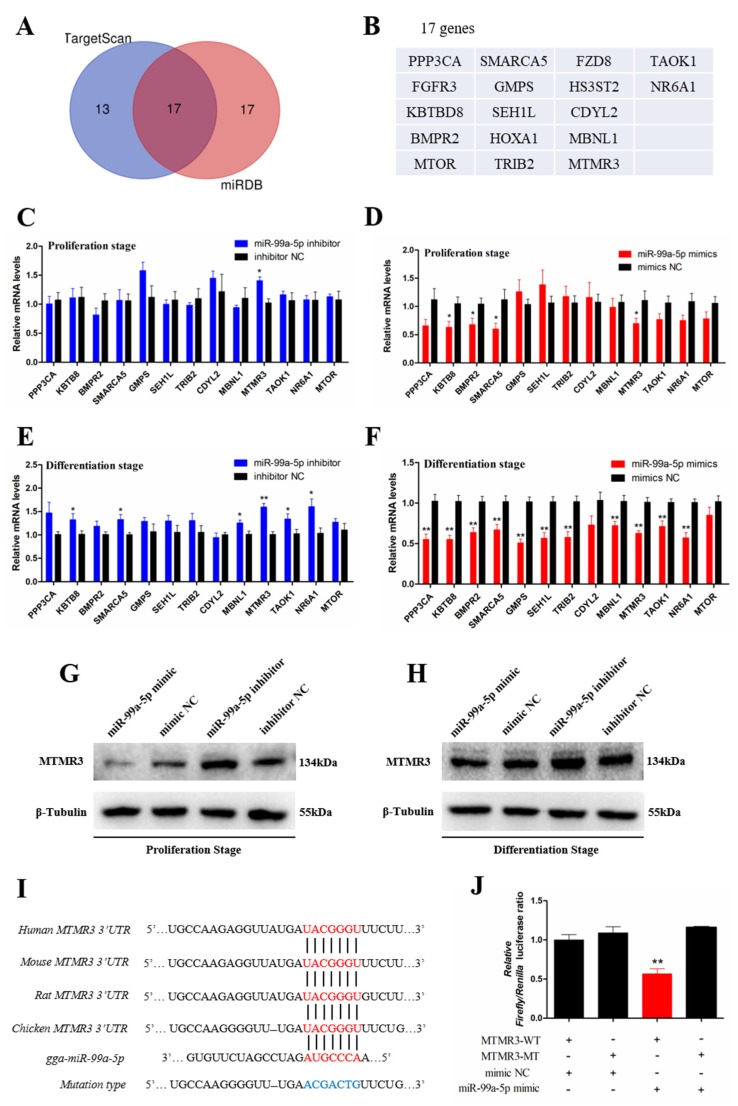
Target gene scanning revealed that miR-99a-5p directly target the myotubularin-related protein 3 (MTMR3) gene. (**A**) Venn analysis of miR-99a-5p target gene prediction results from TargetScan and miRDB websites. (**B**) Seventeen target genes of miR-99a-5p (the intersection of Venn analysis results) are listed. (**C**,**D**) Thirteen target genes of miR-99a-5p were detected by qRT-PCR in SMSCs after overexpression and inhibition of miR-99a-5p in the proliferation stage. (**E**,**F**) Thirteen target genes of miR-99a-5p were detected by qRT-PCR in SMSCs after overexpression and inhibition of miR-99a-5p in the differentiation stage. (**G**) The protein level of MTMR3 were detected by Western blotting in undifferentiated SMSCs after overexpression and inhibition of miR-99a-5p. (**H**) The protein level of MTMR3 was detected by Western blotting in differentiated SMSCs after overexpression and inhibition of miR-99a-5p. (**I**) The conserved miR-99a-5p binding site in the MTMR3 mRNA 3′ untranslated region (UTR). The seed sequence and mutant sequence in miR-34b-5p are highlighted in red and blue, respectively. (**J**) The dual-luciferase reporter assay was performed in DF-1 cells that co-transfected with wild-type or mutant MTMR3 3′ UTR with miR-99a-5p mimic or mimic NC. Results are shown as mean ± SEM, and the data are representative of at least three independent assays. Student’s *t*-test were used to compare expression levels among different groups. * *p* < 0.05; ** *p* < 0.01.

To determine the mechanism by which miR-99a-5p affects SMSC proliferation and differentiation, the TargetScan website and miRDB website were used to search for target genes of miR-99a-5p. The results showed that 30 target genes were found from the TargetScan website and 34 target genes from the miRDB website. In addition, Venn analysis revealed 17 target genes found in both of these two websites (Figure 4A), such as protein phosphatase 3 catalytic subunit alpha isoform (*PPP3CA)*, *MTOR*, and *MTMR3* (Figure 4B). To investigate which of these target genes is regulated by miR-99a-5p, qRT-PCR was performed in SMSCs with modulation of miR-99a-5p expression at the proliferation stage or differentiation stage. With the exception of four genes fibroblast growth factor receptor 3 (*FGFR3)*, homeobox A1 (*HOXA1)*, frizzled homolog 8 (*FZD8)*, and heparan sulfate glucosamine 3-O-sulfotransferase 2 (*HS3ST2*) with low expression in SMSCs that could not be detected, the results showed that knockdown of miR-99a-5p significantly increased *MTMR3* expression (*p* < 0.05; Figure 4C), whereas overexpression of miR-99a-5p decreased the expression levels of kelch repeat and BTB domain containing 8 (*KBTB8)*, bone morphogenetic protein receptor type-2 (*BMPR2)*, SWI/SNF-related, matrix-associated, actin-dependent regulator of chromatin, subfamily a, member 5 (*SMARCA5)*, and *MTMR3* (*p* < 0.05; Figure 4D) at the proliferation stage. As for the differentiated SMSCs, knockdown of miR-99a-5p raised the mRNA levels of *KBTB8*, *SMARCA5*, muscleblind-like1 (*MBNL1)*, *MTMR3*, TAO kinase 1 (*TAOK1)*, and nuclear receptor subfamily 6 group A member 1 (*NR6A1;* (*p* < 0.05; Figure 4E), whereas its overexpression repressed expression of most of these target genes, except chromodomain Y like 2 (*CDYL2)* and *MTOR* (*p* < 0.05; Figure 4F).

Taking these findings together with the qRT-PCR results, we placed a focus on MTMR3 because it was regulated by miR-99a-5p in both the proliferation stage and the differentiation stage. To further explore whether the protein level of MTMR3 was modified by miR-99a-5p, Western blot assay was performed, the results of which revealed that the protein level of MTMR3 was decreased following miR-99a-5p overexpression, whereas it was increased following miR-99a-5p knockdown, irrespective of whether this was in the proliferation stage or the differentiation stage (Figure 4G,H and Figure S3). To determine the binding site of miR-99a-5p on MTMR3′s 3′ UTR, we performed bioinformatic analysis and found that seven bases on this UTR were complementary to the seed sequence of miR-99a-5p; this site is also conserved across many species (Figure 4I). To confirm the targeting relationship between miR-99a-5p and MTMR3′s 3′ UTR, a dual-luciferase reporter assay was performed; our results confirmed that miR-99a-5p could combine with the site of the wild-type reporter, but not the mutant-type reporter of MTMR3′s 3′ UTR (*p* < 0.01; Figure 4J). These results indicated that miR-99a-5p directly targets the MTMR3 gene.

### 3.5. Knockdown of MTMR3 Facilitates Chicken SMSC Proliferation

**Figure 5:**
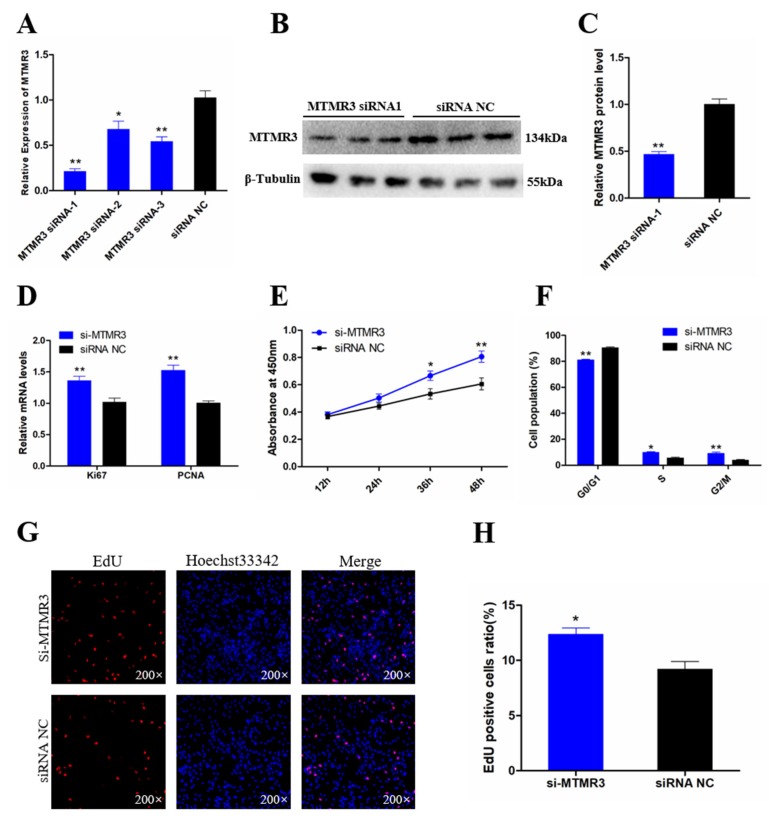
Knockdown MTMR3 facilitates chicken SMSCs proliferation. (**A**) The knockdown efficiency of MTMR3 gene in SMSCs by three small interfering RNAs (siRNAs) were detected by qRT-PCR. (**B**,**C**) The protein expression level of MTMR3 after interference by MTMR3-siRNA1 was detected by Western blotting. (**D**) The mRNA levels of cell proliferation-related genes were detected by qRT-PCR in SMSCs after MTMR3 knockdown. (**E**) CCK-8 assays for SMSCs after MTMR3 knockdown. (**F**) The statistical results of cell cycle analysis for SMSCs after MTMR3 knockdown. (**G**) EdU staining after the transfection of MTMR3-siRNA1 in SMSCs. (**H**) Proliferation rates of chicken SMSCs following MTMR3 knockdown. Results are shown as mean ± SEM and the data are representative of at least three independent assays. Student’s *t*-test were used to compare expression levels among different groups. * *p* < 0.05; ** *p* < 0.01.

To determine the function of MTMR3 in chicken SMSCs, we modulated MTMR3 expression by transfection with three specific siRNAs. We confirmed that the MTMR3 mRNA level was significantly reduced by all three siRNAs compared with negative control siRNA (siRNA NC; *p* < 0.05; Figure 5A); among the three siRNAs, MTMR3 siRNA-1 showed the most effective knockdown effects. The knockdown efficiency of siRNA-1 on MTMR3 protein level was also determined by Western blotting, with the results showing that MTMR3 siRNA-1 significantly decreased the protein level of MTMR3 (*p* < 0.01; Figure 5B, C). Thus, MTMR3 siRNA-1 was chosen for subsequent experiments.

To investigate the effects of MTMR3 on SMSC proliferation, qRT-PCR, CCK-8, flow cytometric, and EdU assays were performed. qRT-PCR analysis showed that knockdown of MTMR3 increased the expression level of cell proliferation-related genes *PCNA* and *Ki67* (*p* < 0.01; Figure 5D). In addition, CCK-8 results revealed that knockdown of MTMR3 improved the cell vitality compared with that in the control group (*p* < 0.05; Figure 5E). Moreover, knockdown of MTMR3 promoted the cell cycle progression of SMSCs into the S and G_2_ phases (*p* < 0.05; Figure 5F). The results of EdU assay showed a similar change of cell proliferation following MTMR3 knockdown (*p* < 0.05; Figure 5G,H). Taken together, these results indicated that MTMR3 plays a negative role in cell proliferation.

### 3.6. Knockdown of MTMR3 Represses Chicken SMSC Differentiation

**Figure 6:**
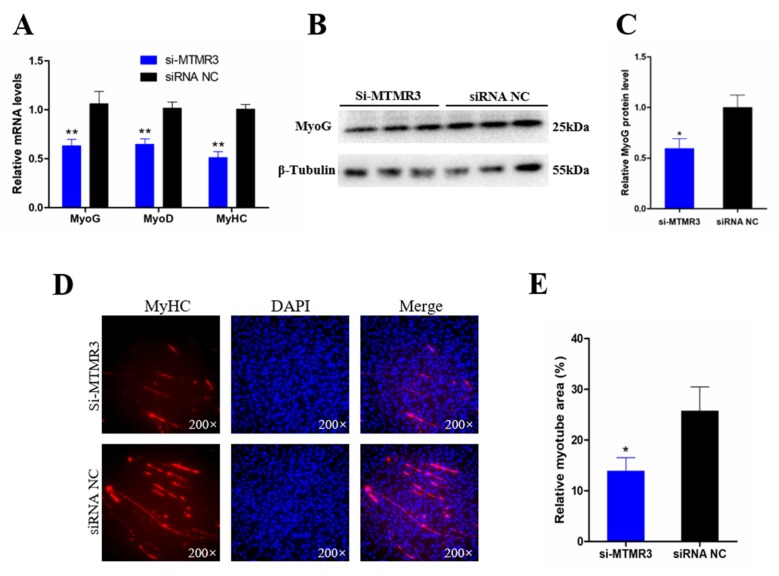
Knockdown MTMR3 represses chicken SMSCs differentiation. (**A**) The mRNA levels of muscle cell differentiation marker genes were detected by qRT-PCR in SMSCs after MTMR3 knockdown. (**B**) Western blotting results of MyoG in SMSCs after transfection with MTMR3 siRNA1. (**C**) The relative protein level of MyoG in SMSCs after inhibition of MTMR3. (**D**) MyHC immunofluorescence staining after the transfection of MTMR3 siRNA1 in SMSCs. (**E**) Relative myotube area of chicken SMSCs following MTMR3 knockdown. Results are shown as mean ± SEM, and the data are representative of at least three independent assays. Student’s *t*-test were used to compare expression levels among different groups. * *p* < 0.05; ** *p* < 0.01.

The ability of MTMR3 to regulate SMSC differentiation was investigated by qRT-PCR, Western blot, and immunofluorescence assays. The qRT-PCR analysis showed that the expression of three muscle differentiation marker genes (*MyoD1*, *MyoG*, and *MyHC*) was significantly decreased by MTMR3 knockdown (*p* < 0.01; Figure 6A). Similarly, MyoG protein level decreased following MTMR3 knockdown (*p* < 0.05; Figure 6B). Moreover, immunofluorescence analysis of MyHC showed that the relative area of myotubes was significantly decreased following MTMR3 knockdown (*p* < 0.05; Figure 6C,D). These results suggested that MTMR3 has a positive role in SMSC differentiation.

## 4. Discussion

It has been widely reported that miR-99a-5p is mainly associated with cancer cell proliferation [11], stem cell self-renewal and differentiation [18,19], and DNA damage response [20], among others. However, to the best of our knowledge, no research has revealed its role in skeletal muscle development and regeneration. In this study, we found that miR-99a-5p is an abundant, brain-enriched miRNA that is differentially expressed during chicken embryonic breast muscle development, which means that it may be involved in skeletal muscle development. Here, a series of experiments were performed to assess the function of miR-99a-5p in chicken SMSC proliferation and differentiation. The results showed that this miRNA promotes the cell cycle progression of SMSCs into the S and G_2_ phases, and also increases the proliferation rate and vitality of SMSCs. In addition, miR-99a-5p inhibits the formation of myotubes by decreasing the expression levels of muscle differentiation marker genes. These findings reveal that miR-99a-5p promotes the proliferation but inhibits the differentiation of chicken SMSCs.

Most studies of miRNAs have focused on their ability to regulate cellular processes by targeting different genes, and have shown that individual miRNAs can have numerous target genes [21,22], as is the case for miR-99a-5p. Specifically, previous reports showed that miR-99a-5p regulates cell biological processes by targeting different genes, such as SMARCA5 [23], MTOR [24], FGFR3 [25], and TRIB2 [10]. To explore which gene(s) was targeted by miR-99a-5p to mediate the proliferation and differentiation of SMSCs, bioinformatic analysis was performed in this study, and 17 candidates were identified from TargetScan and miRDB. Among these genes, two muscle-related genes, PPP3CA and MTOR, drew particular attention here. PPP3CA is an important gene involved in the calcineurin signaling pathway, which initiates skeletal muscle differentiation [26]. Meanwhile, MTOR is a critical gene in the MTOR/P70 S6K signaling pathway, which facilitates skeletal muscle development [27]. Unfortunately, the expression of these two genes showed no significant changes upon miR-99a-5p overexpression or knockdown. However, we found that another gene, MTMR3, exhibited significant responses upon miR-99a-5p overexpression or knockdown, irrespective of whether this was in the proliferation stage or differentiation stage. Using a dual-luciferase reporter assay, we also confirmed that miR-99a-5p directly targets the 3′ UTR of MTMR3. Moreover, a previous report highlighted that miR-99a-5p targets the same site of MTMR3 to regulate human oral cancer cell migration and invasion [28]. Thus, we confirmed that MTMR3 is the most likely target gene of miR-99a-5p during chicken SMSC proliferation and differentiation. As for the issue of whether miR-99a-5p targets other genes during chicken skeletal muscle development, more research is required.

MTMR3 is a member of the myotubularin-related protein family, which is a group of PI3-phosphatases that regulate a range of physiological and biological phenomena, including cell apoptosis and autophagy [14,29]. Furthermore, several members of the myotubularin-related protein family, including MTMR7 [30], MTMR8 [31], and MTMR14 [32], have been demonstrated as being involved in skeletal muscle development. However, whether MTMR3 functions in muscle development had not been revealed. Here, we explored the functions of MTMR3 in chicken SMSCs after the knockdown of MTMR3. The results showed that the knockdown of MTMR3 significantly increased the proliferation ability and decreased the differentiation ability of SMSCs, which means that MTMR3 may play a negative role in chicken SMSC proliferation, while having a positive role in their differentiation. Moreover, MTMR3 was shown to exhibit the opposite effects to miR-99a-5p in chicken SMSC proliferation and differentiation.

## 5. Conclusions

**Figure 7:**
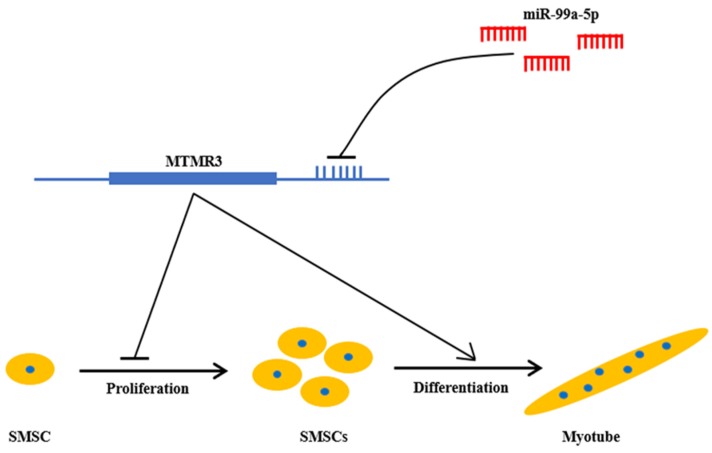
Schematic diagram of miR-99a-5p/MTMR3 axis mediating SMSC proliferation and differentiation.

In this study, we characterized the roles of miR-99a-5p and MTMR3 in the regulation of skeletal muscle development. We confirmed that miR-99a-5p promotes the proliferation but inhibits the differentiation of chicken SMSCs by targeting the MTMR3 gene (Figure 7).

## Figures and Tables

**Table 1:** RNA oligonucleotides in this study.

Name	Sequence (5′–3′)
MTMR3 siRNA-1	CCAGGAGAACACGUAACUUTT
	AAGUUACGUGUUCUCCUGGTT
MTMR3 siRNA-2	CCUAGAGACUGCAGGUAAATT
	UUUACCUGCAGUCUCUAGGTT
MTMR3 siRNA-3	GCGUCUCAACGGUGACUAUTT
	AUAGUCACCGUUGAGACGCTT
siRNA NC	UUCUCCGAACGUGUCACGUTT
	ACGUGACACGUUCGGAGAATT
miR-99a-5p mimic	AACCCGUAGAUCCGAUCUUGUG
Mimic NC	UUGUACUACACAAAAGUACUG
miR-99a-5p inhibitor	CACAAGAUCGGAUCUACGGGUU
Inhibitor NC	CAGUACUUUUGUGUAGUACAA

**Table 2:** Primers for qRT-PCR.

Gene	Primer Sequence (5′–3′)	Length (bp)
*MyoD1*	F: GCCGCCGATGACTTCTATGA	66
	R: CAGGTCCTCGAAGAAGTGCAT	
*MyoG*	F: CGTGTGCCACAGCCAATG	63
	R: CCGCCGGAGAGAGACCTT	
*MyHC*	F: GAAGGAGACCTCAACGAGATGG	138
	R: ATTCAGGTGTCCCAAGTCATCC	
*Ki67*	F: GCAACAACAAGGAGGCTTCG	93
	R: TTCAGGTGCCATCCCGTAAC	
*PCNA*	F: AACACTCAGAGCAGAAGAC	225
	R: GCACAGGAGATGACAACA	
*MBNL1*	F: GTGTCATCGCCTGCTT	133
	R: ATGTTCTTCTGCTGGATCA	
*PPP3CA*	F: CTCATCCTTACTGGCTTCC	219
	R: TCGCTCTTATCTTGTTCCTT	
*KBTBD8*	F: CAAGTCTGTGGAGTGTTATG	237
	R: GCTATGTAGTAGATGGAGTCA	
*BMPR2*	F: TTAGTCCAACAGTCAATCCA	195
	R: AAGTCAGCGGCGTAGT	
*SMARCA5*	F: TTGTCAGAGATGTGTTGTTG	199
	R: GTTAGCAGCAGCCGATT	
*GMPS*	F: CAAGAAGAGCGTCAGAGAA	132
	R: CCATCAGCAACCTTATCCA	
*SEH1L*	F: AGCCAATACAAGCCTACAG	154
	R: GTCAGCATCGCAAGAGT	
*TRIB2*	F: TGACATTGAGCCTAGTTCC	206
	R: CTTCCTTAGCACCATATCCT	
*CDYL2*	F: AGTAATGAATCGCCTGTTGT	140
	R: TGACGCACGCTGTATCT	
*TAOK1*	F: GGAGGAGGAAGAGGAACA	106
	R: TTGGCTACTGGCACTGA	
*NR6A1*	F: ATGGAGCAACAATGGAGAC	203
	R: CCGAGTGAGCAGAATAGC	
*MTOR*	F: GGAATGAACCGTGATGAC	121
	R: GCTGCTGCTGAGTGAT	
*MTMR3*	F: TGTAGGACGAGCAGAAGA	227
	R: GGCGGATGGCATTGTT	
*GAPDH*	F: CCAGAACATCATCCCAGCGTC	136
	R: ACGGCAGGTCAGGTCAACAA	
*miR-99a-5p*	F: AACCCGTAGATCCGATCTTGTG	/
	R: CAGGTCCAGTTTTTTTTTTTTTT	
*U6*	F: GGGCCATGCTAATCTTCTCTGTA	/
	R: CAGGTCCAGTTTTTTTTTTTTTT	

## Supplementary Materials

The following are available online at https://www.mdpi.com/2073-4425/11/4/369/s1. Figure S1: Desmin immunofluorescence staining of chicken SMSCs, Figure S2: (A) CCK-8 assay results of chicken SMSCs which cultured in GM. (B) MyHC immunofluorescence staining of chicken SMSCs of which cultured in GM or DM. (C) The expression of three muscle cell differentiation marker genes in chicken SMSCs of which cultured in GM or DM, Figure S3: (A) The relative protein level of MTMR3 in undifferentiated SMSCs after overexpression and inhibition of miR-99a-5p. (B) The relative protein level of MTMR3 in differentiated SMSCs after overexpression and inhibition of miR-99a-5p.
