# Supplementary material for: miR-99a-5p Regulates the Proliferation and Differentiation of Skeletal Muscle Satellite Cells by Targeting MTMR3 in Chicken
Source: Genes (Basel). 2020 Mar 29;11(4):369. doi: 10.3390/genes11040369 (PMC7230175; doi:10.3390/genes11040369)
Supplement: Supplementary file 1 [file genes-11-00369-s001.pdf]

# miR-99a-5p Regulates the Proliferation and Differentiation of Skeletal Muscle Satellite Cells by Targeting MTMR3 in Chicken

**Xinao Cao[1], Shuyue Tang[1], Fei Du[1], Hao Li[1], Xiaoxu Shen[2], Diyan Li[2], Yan Wang[2], Zhichao Zhang[1], Lu Xia[2], Qing Zhu[2], Huadong Yin[2*]**

1 College of Animal Science and Technology, Sichuan Agricultural University, Chengdu, Sichuan 611130, PR China;

2 Farm Animal Genetic Resources Exploration and Innovation Key Laboratory of Sichuan Province, Sichuan Agricultural University, Chengdu, Sichuan 611130, PR China.

* Correspondence: yinhuadong@sicau.edu.cn (H.Y.)

## Supplementary Figures

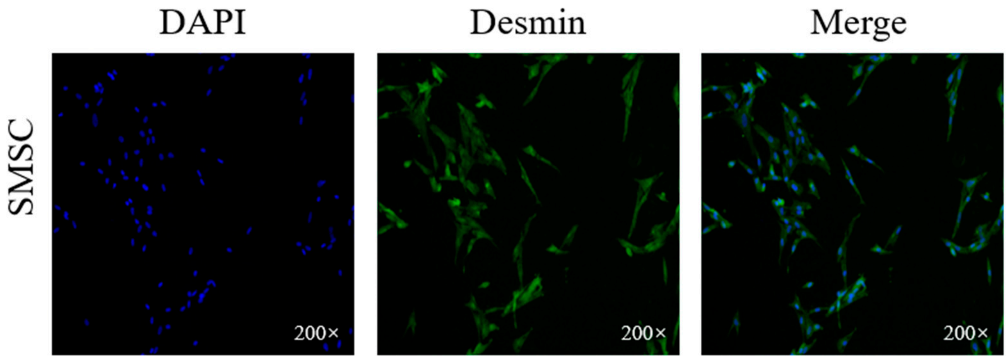


**Figure S1.** Desmin immunofluorescence staining of chicken SMSCs.

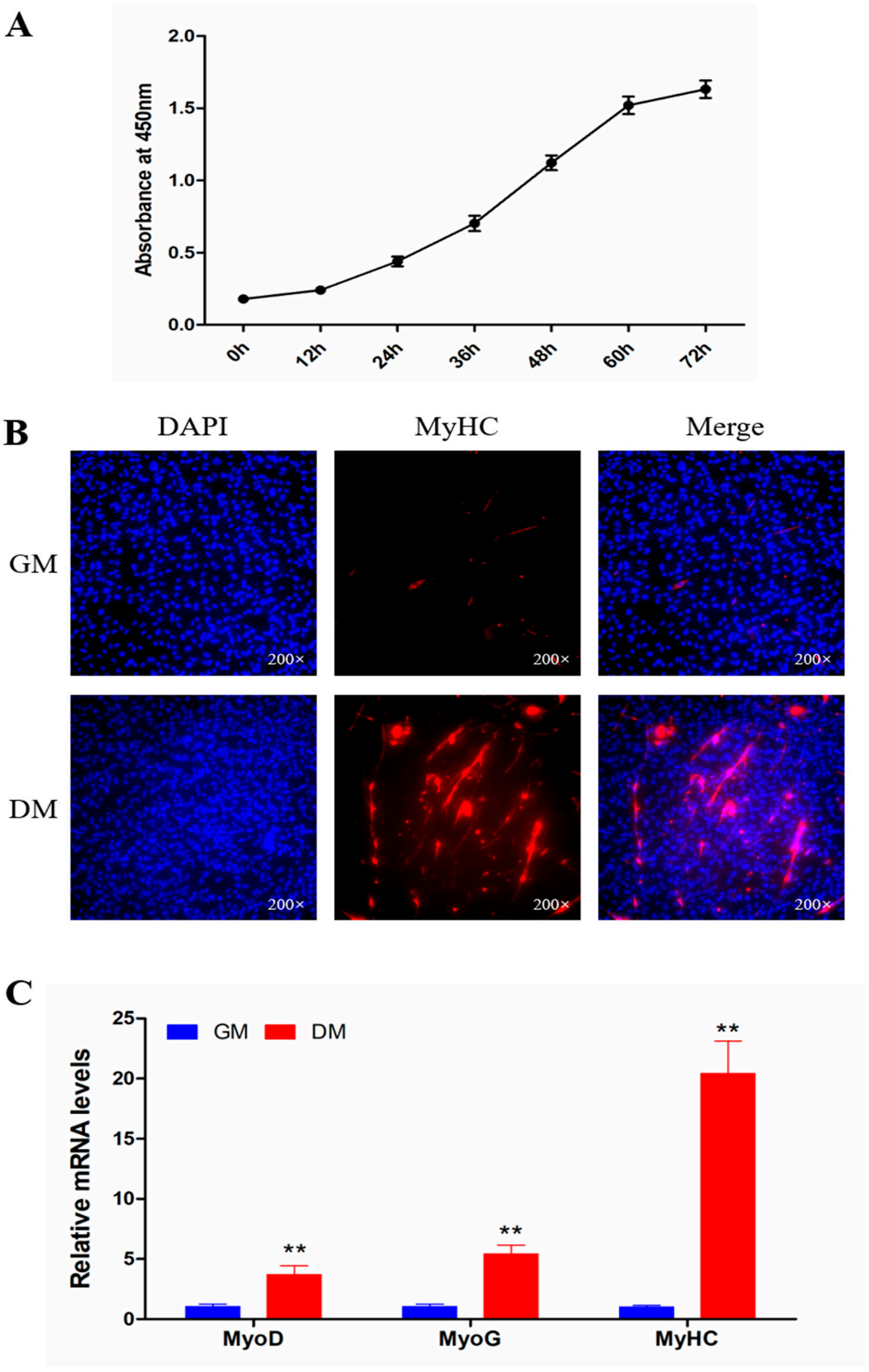


**Figure S2. (A)** CCK-8 assay results of chicken SMSCs which cultured in GM. **(B)** MyHC immunofluorescence staining of chicken SMSCs of which cultured in GM or DM. **(C)** The expression of three muscle cell differentiation marker genes in chicken SMSCs of which cultured in GM or DM.

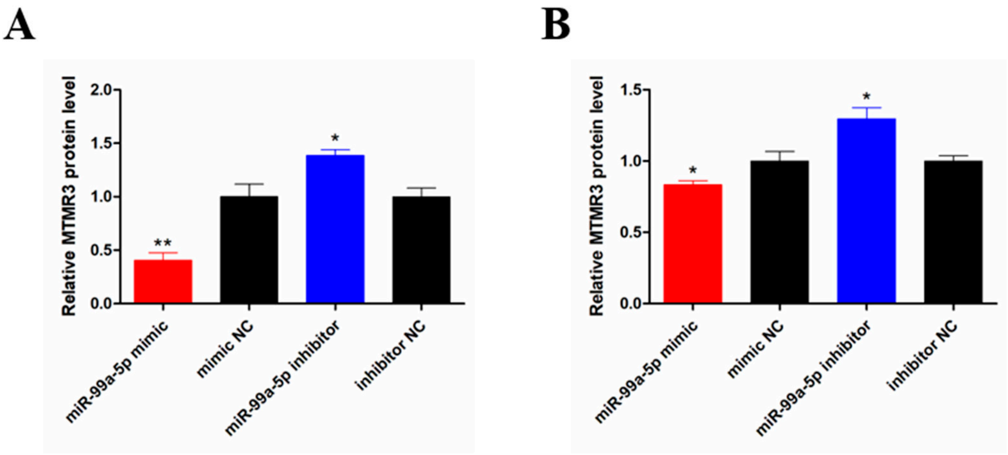


**Figure S3. (A)** The relative protein level of MTMR3 in undifferentiated SMSCs after overexpression and inhibition of miR-99a-5p. **(B)** The relative protein level of MTMR3 in differentiated SMSCs after overexpression and inhibition of miR-99a-5p.
